# Myxoglobulosis of the appendix presenting as acute appendicitis

**DOI:** 10.1093/jscr/rjad624

**Published:** 2023-11-20

**Authors:** Khalid Abuaagla, Shehla Faridoon, Atef Hassan, Mudather Bafadni, Mohammed A Rabih, Abdelhakim Alsaadi

**Affiliations:** General Surgery Department, Prince Abdelmohsin Hospital, Alula 70, Al Ula 43543, Saudi Arabia; General Surgery Department, Prince Abdelmohsin Hospital, Alula 70, Al Ula 43543, Saudi Arabia; General Surgery Department, Prince Abdelmohsin Hospital, Alula 70, Al Ula 43543, Saudi Arabia; General Surgery Department, Prince Abdelmohsin Hospital, Alula 70, Al Ula 43543, Saudi Arabia; General Surgery Department, Prince Abdelmohsin Hospital, Alula 70, Al Ula 43543, Saudi Arabia; General Surgery Department, Prince Abdelmohsin Hospital, Alula 70, Al Ula 43543, Saudi Arabia

**Keywords:** myxoglobulosis, appendiceal mucocele, appendicitis

## Abstract

Myxoglobulosis is a rare form of appendiceal mucocele characterized by mucoid material inside the appendix that resembles fish eggs. It is usually asymptomatic and diagnosed incidentally, but it can also present as a surgical abdomen, which can create a diagnostic dilemma. This case report presents a 37-year-old male patient with features suggestive of acute appendicitis. A computed tomography scan of the abdomen showed features of appendiceal mucocele. The patient underwent appendicectomy through a lower midline incision. The macroscopic finding was myxoglobulosis, and the patient had an uneventful postoperative course. The histopathology report of the appendix confirmed the diagnosis.

## Introduction

Appendiceal mucocele is a rare disease in which the appendix fills with mucus. It may be associated with neoplastic or non-neoplastic pathologies. There are four histological groups of appendiceal mucocele: mucosal hyperplasia, simple or retention cysts, mucinous cystadenomas, and mucinous cystadenocarcinomas [1]. In appendectomy specimens, the incidence of appendiceal mucocele is 0.2 to 0.3% [2]. Approximately one-third of cases present with acute appendicitis; however, most cases are discovered incidentally [3]. On cross-sectional imaging, appendiceal mucocele often appears as a low-attenuation, round, and well-encapsulated cyst in the right lower quadrant. Features such as wall irregularity and thickening of soft tissue suggest neoplasia [1]. Radiological investigations alone cannot provide a reliable diagnosis of appendiceal mucocele, so surgical excision without capsular disruption is recommended [2, 4]. Myxoglobulosis, or caviar appendix, is an uncommon type of mucocele. Its characteristic feature is the presence of opaque globules that resemble fish eggs or frogspawns because they contain mucoid material [5, 6]. This case report describes a patient with myxoglobulosis of the appendix presenting with acute appendicitis.

## Case report

A 37-year-old male shepherd presented with features suggestive of acute appendicitis. He had one day of lower quadrant abdominal pain, which was dull aching in nature, started peri umbilically, and then shifted to the right iliac fossa. The pain was associated with nausea, loss of appetite, and two episodes of vomiting. He had no fever or change in bowel habits. He had a previous history of recurrent right iliac fossa pain, mild in nature, one year ago. Abdominal examination revealed deep right iliac fossa tenderness with negative rebound tenderness. Blood tests were unremarkable, apart from a high leukocyte count of 13 000.

**Figure 1:**
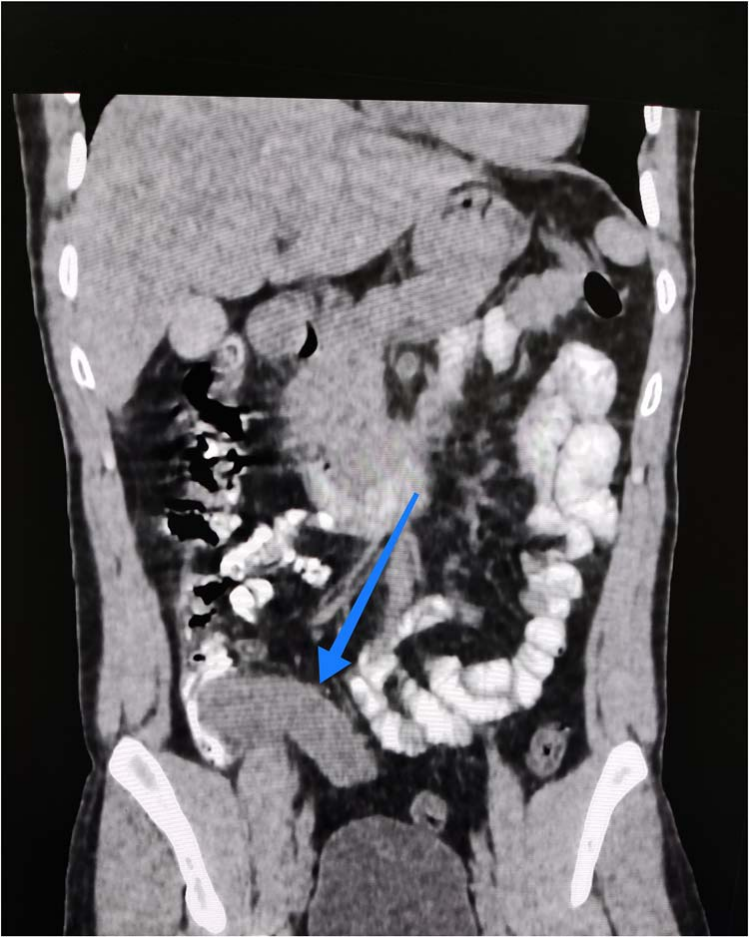
Coronal view of abdominal contrast CT showing the oblong structure originating from the medial side of the caecum and extending down to the pelvis.

Abdominal ultrasound reported a mass with a cystic component at the right iliac fossa with unclear margins, suggestive of an appendicular abscess. Consequently, a computed tomography (CT) abdomen with oral and IV contrast was requested. It showed an oblong, fluid-filled structure originating from the medial side of the caecum, 14 cm long and 3 cm wide, with a wall thickness of 5.3 mm, extending down to the pelvis (Fig. 1). This resulted in a provisional diagnosis of either an appendiceal mucocele or mucinous appendiceal neoplasm.

**Figure 2:**
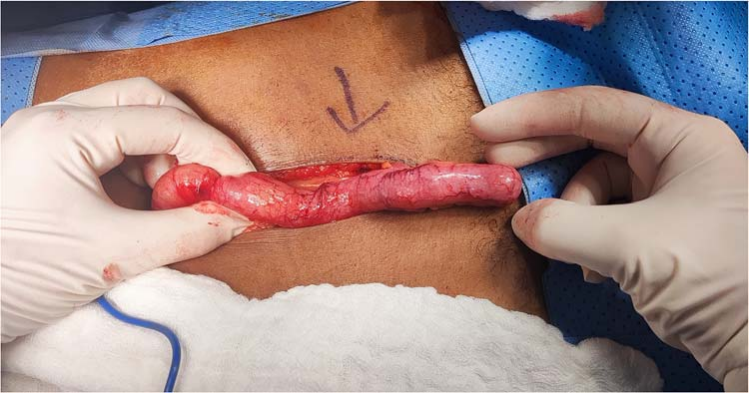
Intraoperative view of the mucocele.

Laparotomy exploration took place under general anaesthesia through a lower midline incision. The operative finding was a mobile appendiceal mucocele, soft adhesion, and a mobile caecum (Fig. 2). An appendectomy was then performed without spillage, with the appendix base secured with a transfixing stitch and buried with a purse string suture. A thorough abdominal examination followed. There were no ascites, lymph nodes, peritoneal, or liver deposits noticed.

**Figure 3:**
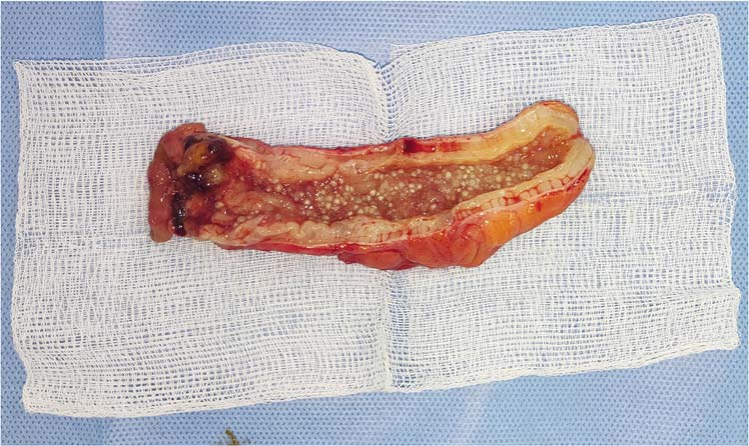
Appendiceal specimen showing fish egg shape of myxoglobulosis.

On gross examination, the removed appendix contained small, rounded white globules a few millimetres in diameter (Fig. 3). Postoperatively, the patient experienced no complications and was discharged in good condition after 3 days. He was seen at the outpatient clinic two weeks later and had his surgical clips removed.

Histopathology reports showed acute appendicitis and the white globules as mucinous material, with the final diagnosis of acute appendicitis with myxoglobulosis of the appendix.

## Discussion

Appendiceal myxoglobulosis, a subtype of appendiceal mucocele, is characterized by mucinous, often calcified, pearl-shaped globules in the lumen [6, 7]. It was first described by Latham [5] in a postmortem case in 1897. An appendiceal mucocele is estimated to occur in 0.2–0.3% of appendectomy specimens, with myxoglobulosis constituting 0.35–0.8% of all mucoceles [8]. The cause of myxoglobulosis is unknown. It usually occurs in the sixth or seventh decade of life, with a slight female predominance [5]. Myxoglobulosis is usually diagnosed incidentally during autopsies or laparotomies for another reason [5, 6]. Although appendix perforation is a frequently reported complication, peritonitis or pseudomyxoma peritonei are typically thought to be the most common complications [8].

### Pathogenesis

In various hypotheses, the formation of a core, which is a nidus for mucin deposition, is thought to initiate the pathogenesis of the globules. Some authors suggest that small mucinous masses might develop in dilated glandular crypts from bacterial and necrotic epithelial debris [8, 9]. In Lubin and Berle’s [10] most accepted proposal, the core of each globule is a necrotic fragment of granulation tissue that has broken loose from the appendix wall.

### Diagnosis

The diagnosis of myxoglobulosis cannot be made based on specific laboratory findings. Radiology studies, especially CT scans, can suggest a diagnosis, but pathologic confirmation of mucin globules is required [11]. When found on a CT scan, an encapsulated, hypoattenuating cystic mass, often with punctate calcification, can be pathognomonic of mucocele with myxoglobulosis [12].

### Treatment

All appendiceal mucoceles should undergo early surgical resection to exclude mucinous neoplasms and prevent spontaneous rupture [4]. Prevention of iatrogenic rupture requires careful handling of the intact appendix during intraoperative procedures. The optimal surgical procedure is a matter of controversy. Standard appendectomy is the initial procedure [13]. It is important to follow the pathological diagnosis in determining the extent of surgery. If mucosal hyperplasia or mucinous cystadenoma is present, simple appendectomy is sufficient. If a cystadenocarcinoma is present, it is recommended to perform an ileocecal resection or a right hemicolectomy with lymph node dissection [4]. Several factors need to be considered, including the tumour size, location, mucin content, involvement of the caecum and ileum, lymph node involvement, margin status, and the final pathology report [2].

### Open vs. laparoscopic surgery

The suitability of open surgery over laparoscopic surgery is continuously debated. Due to more meticulous handling, an open approach reduces the chances of rupture. Palpating the tumour during surgery and determining the optimal resection is possible. Furthermore, if detected during a diagnostic laparoscopy, the procedure is better converted to an open procedure [14]. Fewer than 100 cases of appendiceal myxoglobulosis have been reported in the literature. To our knowledge, this is the first reported case in Saudi Arabia.
